# Lower nutritional state and foraging success in an Arctic seabird despite behaviorally flexible responses to environmental change

**DOI:** 10.1002/ece3.9923

**Published:** 2023-04-20

**Authors:** Alyssa Eby, Allison Patterson, Graham Sorenson, Thomas Lazarus, Shannon Whelan, Kyle H. Elliott, H. Grant Gilchrist, Oliver P. Love

**Affiliations:** ^1^ Department of Integrative Biology University of Windsor Windsor Ontario N9B 3P4 Canada; ^2^ Department of Natural Resource Sciences McGill University Ste Anne‐de‐Bellevue Quebec H9X 3V9 Canada; ^3^ Environment and Climate Change Canada National Wildlife Research Centre 1125 Colonel By Drive, Raven Road Ottawa Ontario K1A OH3 Canada; ^4^ Present address: Atlantic Region Office Birds Canada Sackville New Brunswick E4L 1G6 Canada

**Keywords:** Arctic, climate change, daily energy expenditure, foraging flexibility, foraging success: nutritional biomarkers, sea ice, thick‐billed murre

## Abstract

The degree to which individuals adjust foraging behavior in response to environmental variability can impact foraging success, leading to downstream impacts on fitness and population dynamics. We examined the foraging flexibility, average daily energy expenditure, and foraging success of an ice‐associated Arctic seabird, the thick‐billed murre (*Uria lomvia*) in response to broad‐scale environmental conditions at two different‐sized, low Arctic colonies located <300 km apart. First, we compared foraging behavior (measured via GPS units), average daily energy expenditure (estimated from GPS derived activity budgets), and foraging success (nutritional state measured via nutritional biomarkers pre‐ and post‐ GPS deployment) of murres at two colonies, which differ greatly in size: 30,000 pairs breed on Coats Island, Nunavut, and 400,000 pairs breed on Digges Island, Nunavut. Second, we tested whether colony size within the same marine ecosystem altered foraging behavior in response to broad‐scale environmental variability. Third, we tested whether environmentally induced foraging flexibility influenced the foraging success of murres. Murres at the larger colony foraged farther and longer but made fewer trips, resulting in a lower nutritional state and lower foraging success compared to birds at the smaller colony. Foraging behavior and foraging success varied in response to environmental variation, with murres at both colonies making longer, more distant foraging trips in high ice regimes during incubation, suggesting flexibility in responding to environmental variability. However, only birds at the larger colony showed this same flexibility during chick rearing. Foraging success at both colonies was higher during high ice regimes, suggesting greater prey availability. Overall, murres from the larger colony exhibited lower foraging success, and their foraging behavior showed stronger responses to changes in broad‐scale conditions such as sea ice regime. Taken together, this suggests that larger Arctic seabird colonies have higher behavioral and demographic sensitivity to environmental change.

## Graphical abstract

Recent climate‐induced changes in sea‐ice dynamics are impacting Arctic marine ecosystems, further exacerbating the effects of colony size on foraging behavior. To assess the adaptive capacity of Arctic seabirds to cope with changing sea ice dynamics, our study investigates the foraging behavior and resulting foraging success of thick‐billed murres under different sea ice regimes at two different‐sized colonies. We found that thick‐billed murres at both colonies had lower foraging success under low ice regimes and that thick‐billed murres at a larger colony had overall lower foraging success suggesting larger colonies in the Canadian Arctic may be more susceptible to environmental change.

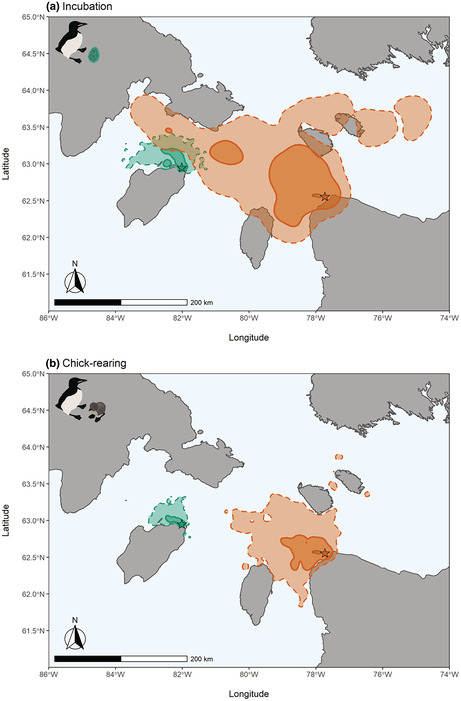

## 1 INTRODUCTION

Colonial breeding is widespread in animals and confers both advantages and costs (Rolland et al., 1998). Dense, large breeding aggregations provide mating opportunities, protection from predators, and information sharing of foraging hotspots (Davoren et al., 2003; Ward & Zahavi, 1973). Despite the increased foraging efficiency associated with information sharing, large breeding aggregations can also negatively impact foraging efficiency, as larger colonies deplete resources surrounding the colony at a higher rate, leading to larger foraging ranges (Ashmole's halo hypothesis) (Ashmole, 1963; Cairns, 1989; Elliott, Woo, Gaston, Benvenuti, et al., 2009; Storer, 1952). Larger foraging ranges can in turn decrease foraging success, offspring‐provisioning rates, offspring‐growth rates, and fitness, effectively limiting population growth (Ashmole, 1963; Cairns, 1989; Elliott, Woo, Gaston, Benvenuti, et al., 2009; Storer, 1952). In addition to colony size, environmental conditions and variability can also impact animal foraging behavior and movement by altering prey availability and abundance (St. John Glew et al., 2019). However, while changing environmental conditions will likely affect colonies of different sizes at varying magnitudes via direct mechanisms (e.g., prey availability and abundance), we know little about how and why environmental variation impacts fitness via more complex indirect mechanisms (Barbraud & Weimerskirch, 2003).

As physiological metrics represent a link between the individual and its environment, they are often thought of as useful markers and regulators of life history investment and trade‐offs (Hennin et al., 2018; Ricklefs & Wikelski, 2002; Zera & Harshman, 2001). Simultaneous measurements of multiple nutritional biomarkers (e.g., energetic metabolites and hormones; Madliger et al., 2018) may improve assessment of the relative costs and benefits of different foraging strategies (Wilmers et al., 2015) because the biomarkers provide information on foraging profitability (Guglielmo et al., 2005; Morales et al., 2020; Storey et al., 2017; Williams et al., 1999). Due to logistical or financial constraints, nutritional biomarkers are often measured at a single time point, giving only a snapshot of nutritional state (Dunphy et al., 2020). However, when paired with biologger deployments, the need to recapture individuals to retrieve devices provides an opportunity to obtain an additional measure of nutritional biomarkers and pair them with observations of foraging behavior. Multiple sampling events can be used to assess foraging success, the relative success of foraging trips over the deployment period, by comparing pre‐ and post‐foraging levels of nutritional biomarkers (Tarroux et al., 2020). Pairing foraging behavior (measured via biologging) and environmental conditions (measured via satellite imagery) with changes in nutritional biomarkers can thus provide a direct representation of how variation in environmental conditions and foraging behavior impacts foraging success (Table 1).

**TABLE 1:** Summary of nutritional biomarkers and their biological interpretation.

Nutritional biomarker	Name	Biological interpretation
Mass	Mass	Higher mass indicates a higher nutritional state and greater foraging success (Gaston & Hipfner, 2006b; Hipfner et al., 2006; Storey et al., 2017; Tarroux et al., 2020)
Baseline corticosterone	bCORT	Plays an integral role in the maintenance of homeostasis and the regulation of energy expenditure—lower levels indicate a higher nutritional state and greater foraging success (Benowitz‐Fredericks et al., 2008; McEwen & Wingfield, 2003)
Non−esterified fatty acids	NEFA	During periods of high energy demand, where energy output is greater than energy intake, hydrolysis of triglycerides from adipose tissue form non−esterified fatty acids—lower levels indicate a higher nutritional state and greater foraging success (Jenni‐Eiermann & Jenni, 1994; Williams & Buck, 2010)
Beta‐hydroxybutyrate	B‐OH	During periods of fasting or body mass loss, beta‐hydroxybutyrate is synthesized from free fatty acids to be used as fuel for tissues—lower levels indicate a higher nutritional state and greater foraging success (Anteau & Afton, 2008; Cherel et al., 1988; Guglielmo et al., 2005)
Plasma triglycerides	TRIG	The storage form of fatty acids and thus can be used as an indicator of fat deposition—higher levels indicate a higher nutritional state and greater foraging success (Dietz et al., 2009; Gerson & Guglielmo, 2013)

Rising Arctic temperatures have increased interannual variability of sea ice dynamics and reduced Arctic sea ice extent (Mioduszewski et al., 2019). Pagophilic (ice‐obligate or ice‐associated) species experience the greatest negative impacts from sea ice decline and fluctuations through both direct and indirect processes (Macias‐Fauria & Post, 2018). Loss of sea ice will have direct and detrimental effects on species that use ice for hunting (e.g., polar bears *Ursus maritimus*) or breeding (e.g., ringed seals *Phoca hispida* and bearded seals *Erignathus barbatus*; Laidre, Stirling, et al., 2008; Macias‐Fauria & Post, 2018). For example, as a result of earlier sea ice retreat, subpopulations of polar bears in Nunavut, Canada, are now spending more time on land (Smith et al., 2010), resulting in lower body condition and lower reproductive success (Laidre et al., 2020), and changes in foraging behavior (Iverson et al., 2014; Jagielski, Dey, Gilchrist, Richardson, Love, & Semeniuk, 2021; Jagielski, Dey, Gilchrist, Richardson, & Semeniuk, 2021). Indirectly, changing sea ice dynamics can cause phenological mismatches within Arctic marine food webs that decrease the prey available for higher trophic level species (e.g., whales, seals, seabirds; Macias‐Fauria & Post, 2018). For instance, the relatively rapid response of primary producers to reduced sea ice cover in Svalbard, Norway, led to a reduction in the breeding success of two seabird species: the little auk (*Alle alle*) and thick‐billed murres (*Uria lomvia*) (Ramírez et al., 2017). Similarly, murre chicks in the low Canadian Arctic grew at slower rates when sea ice break‐ups were earlier (Gaston et al., 2009). Given that climate change can decrease food available to Arctic predators, colonial species—and especially larger colonies—may be particularly sensitive to environmental change.

Geographically widespread seabird species such as thick‐billed murres (hereafter referred to as murres) that range from the northern Atlantic and Pacific to the high Arctic (Gaston & Hipfner, 2020) experience a range of environmental conditions across populations (Laidre, Heide‐Jørgensen, et al., 2008). For example, murres nesting at high Arctic sites benefit from years with less sea ice due to increased access to prey items resulting in higher breeding success (Gaston, Gilchrist, & Mallory, 2005). Conversely, murres breeding at low Arctic sites experienced a prey switch in the late 1990s corresponding to a decline in sea ice extent, leading to reduced chick‐provisioning and chick growth rates (Gaston et al., 2003; Gaston & Elliott, 2014; Gaston, Gilchrist, & Hipfner, 2005). As earlier sea ice retreat is beneficial to murres breeding in the high Arctic but disadvantageous to murres breeding in the low Arctic, we may see a contraction of murre breeding range, as murres shift northward to breeding exclusively at high Arctic sites.

Furthermore, recent findings suggest environmental conditions during the nonbreeding season (overwintering and prebreeding areas) are one factor driving major differences in population trends of murres observed across the Atlantic, in addition to mortality caused by hunting and oil spills (Frederiksen et al., 2016, 2021). Rapid population declines are occurring in eastern Atlantic populations (breeding colonies in Iceland, Svalbard, and southwest Greenland) that overwinter in waters around southwest Greenland and Iceland (Frederiksen et al., 2016, 2021). Meanwhile, western Atlantic populations (breeding colonies in Canada and northwest Greenland), which overwinter in waters off Labrador and Newfoundland, remain stable (Frederiksen et al., 2016, 2021). In addition to the effect of geographic location and environmental change on seabird breeding success in a changing Arctic, the overall size of breeding colonies is also expected to play an interactive role, as murre colonies can range in size across several orders of magnitude, from fewer than 500 breeding pairs (Merkel et al., 2014) to more than 800,000 breeding pairs (Hickey & Craighead, 1977), and recent work has shown that murre foraging range scales to colony size with an exponent of 0.33 (Patterson et al., 2022).

Here we use a multiyear integrative field study to examine the drivers of inter‐ and intracolony variation in foraging behavior and success in an Arctic breeding, colonial seabird facing rapid environmental change. Specifically, our aims were to (i) examine intercolony variation in foraging behavior, energy expenditure, and foraging success during two breeding stages at two different sized colonies experiencing similar environmental conditions, (ii) assess whether murres exhibited foraging flexibility in response to broad‐scale environmental variability, and (iii) determine whether environmentally induced foraging flexibility impacted foraging success. As prey depletion rates are expected to be directly related to colony size (Ashmole, 1963; Cairns, 1989; Elliott, Woo, Gaston, Benvenuti, et al., 2009; Gaston et al., 2007), we expected murres from large and small colonies to differ in their foraging behavior. We predicted that at low Arctic colonies years with lower sea ice concentration and higher sea surface temperatures would have lower prey abundance (as chick growth rates have been previously recorded as slower when sea ice retreat is earlier; Gaston et al., 2009), resulting in increased search time for prey items, and therefore longer foraging trips. Given that murres at larger colonies need to travel farther distances and incur higher foraging costs, we expected a lower nutritional state among birds at larger colonies because they are under greater energetic constraints and closer to their physiological limits. Although we expected to find flexibility in foraging behavior to allow murres across colony sizes to respond to environmental change, we also expected stronger negative downstream effects of environmental change on the success of this foraging flexibility for birds breeding at a larger colony. We then assess how interactions between colony size and behavioral responses to environmental variability may affect the resilience of breeding populations to Arctic climate change.

## 2 METHODS

### 2.1 Study sites: Coats Island and Digges Island, Nunavut

We conducted fieldwork at two murre colonies located within the Hudson Strait‐Northern Hudson Bay Narrows region of the Eastern Canadian Arctic: Coats Island, NU (West colony, 62.95° N, 82.01° W and East colony, 62.95° N, 81.98° W; sampled from 2017 to 2019), and Digges Sound, NU (Digges Island, 62.55°N, 77.73°W and Cape Wolstenholme, 62.55°N, 77.54°W; sampled from 2014 to 2016, referred to hereafter as Digges Island). Although the sites are only 220 km apart, they vary greatly in colony size. Coats Island hosts 30,000 breeding pairs and 400,000 pairs breed on Digges Island (Gaston et al., 2013). Murres nest on rocky cliff ledges at both sites, but the limited cliff extent (<2 km of cliff habitat) at Coats Island limits population size, while nesting habitat is apparently unlimited on the 12 km of cliffs at Digges Island (Gaston et al., 1993).

### 2.2 Environmental conditions

To quantify environmental variation, we used sea ice concentration and sea surface temperature (SST) obtained through remote sensing (global ocean Operational SST and Ice Analysis, OSTIA; Copernicus Marine Environment Monitoring Service). Murres are associated with sea ice (Bonnet‐Lebrun, Larsen, Frederiksen, et al., 2021; Bonnet‐Lebrun, Larsen, Thórarinsson, et al., 2021; Cusset et al., 2019; Laidre, Heide‐Jørgensen, et al., 2008; LeBlanc et al., 2019) and both sea ice concentration and SST predict breeding phenology at Coats Island (Gaston & Elliott, 2014). Furthermore, these two environmental variables are known to influence local prey availability (Gaston & Elliott, 2014; Laidre, Heide‐Jørgensen, et al., 2008). We calculated daily mean sea surface temperature and sea ice concentration within the radius of the maximum foraging range of each colony (Coats Island = 130 km; Digges Island = 300 km) throughout the breeding season, from the period of June 15th (prior to egg‐laying) to August 15th (when chicks fledge and sea ice is typically no longer present within Hudson Strait and Hudson Bay).

### 2.3 Murre field sampling and GPS deployment

We conducted all fieldwork under a University of Windsor Animal Utilization Project Proposal (15‐04), a McGill Animal Use Protocol (2015‐7599), and Environment and Climate Change Canada Animal Care and Collection permits (NUN‐SCI‐14‐11, EC‐PN‐14‐017, EC‐PN‐15‐017). We captured adult murres during incubation and chick rearing using a noose pole at nest sites. We blood sampled murres within 3 minutes of capture (to ensure the measurement of baseline physiology—see below), collecting 1–2 mL of whole blood from the brachial or jugular vein using either a 26‐guage needle, heparinized capillary tube, and heparinized Eppendorf tube (Digges Island) or a 25‐guage needle, 3 mL syringe, and heparinized vacutainer (Coats Island). We kept blood samples on ice, for a maximum 8 h, and then centrifuged for 5–10 min at 2000 *g* to separate plasma from red blood cells. We then transferred plasma into cryovials and stored at −80°C until laboratory assays. We used a smear of red blood cells for molecular sexing, following Elliott et al. (2010). We used behavioral sexing on Coats Island for murres that were not molecularly sexed; murres at Coats Island have sex‐specific foraging behavior, where males attend the nest during the day and forage at night, whereas females attend the nest during the evening and forage during the day (Elliott et al., 2010). If a murre was consistently at the colony between 23 h 30 min and 3 h 30 min it was classified as female, whereas if a bird was at the colony between 11 h 30 min and 15 h 30 min it was classified as male (previously shown to be 100% accurate compared to molecular sexing, unpubl. data, K. Elliott).

After blood sampling, we banded murres for individual identification and measured body mass (g). We attached GPS devices (CatTraQ™, Catnip Technologies, 18 g, 1.90% of body mass; Uria‐100™, Ecotone, 16 g, 1.68% of body mass; AXY‐Depth™, Technosmart, 6.5 g, 0.68% of body mass, AxyTrek™, Technosmart, 18 g, 1.90% of body mass) to murres' dorsal feathers (Paredes et al., 2005). We began retrieval efforts 1–4 days after deployment (devices retrieved between 1–17 days; Digges mean ± SE = 4.1 ± 0.16 days; Coats mean ± SE = 2.2 ± 0.05 days). Upon recapture, we collected a second blood sample, re‐measured body mass, and removed the GPS device.

Breeding plots at Coats Island were monitored daily to estimate lay date, hatch date, and fledge date at each nest site from 2014 to 2019 (following Gaston et al., 2009). Median lay date and hatch dates were then calculated for each year. Since infrastructure limitations at Digges Island precluded nest monitoring, median lay dates, and hatch dates from Coats Island were used from respective years as estimates. In the four years when monitored simultaneously, Digges' median hatch date was 1 to 4 days (mean ± SE = 3 ± 0.7 days) later than Coats (unpubl. data, K. Elliott). We therefore used median hatch date at Coats plus three days to estimate Digges median hatch date. Median lay dates and hatch dates were overlaid on sea ice concentration and sea surface temperature graphs to illustrate the impact of environmental conditions on breeding phenology, as previously noted at Coats Island (Whelan et al., 2022).

### 2.4 Foraging metrics and average daily energy expenditure

We processed the GPS data and extracted foraging metrics in *R* (version 4.03, R Core Team, 2020). We considered murres to be on a foraging trip if they were further than 1 km away from the colony, to filter out locations associated with preening and socializing in the splashdown area adjacent to the colony (Brisson‐Curadeau et al., 2018; Burger, 1997; Elliott, Bull, Gaston, & Davoren, 2009). Within a trip, we considered murres to be flying if ground speed was above 14.4 km/h and swimming if below 14.4 km/h. To summarize foraging trips, we calculated the maximum distance traveled (the furthest distance from the colony; km), total distance traveled (km), and trip duration (hours). To summarize activity over the entire deployment period for each individual, we calculated the maximum trip distance (km), average daily distance traveled (km), mean trip distance (km), mean trip duration (h), and number of trips per day from foraging trips (Table 2). We used the duration a murre spent flying (*T*_Flying;_ h), at the colony (*T*_Colony;_ h), and swimming (*T*_Swimming;_ h) over the deployment period to estimate average daily energy expenditure (DEE; kJ/d) using the equation:

$$\mathrm{DEE}=\left(\frac{32.0\times T_{\text{Colony}}+532.8\times T_{\text{Flying}}+99.0\times T_{\text{Swimming}}}{\text{Deployment Duration}}\right)\times 24,$$

where constants are the amount of energy (kJ) used during each activity estimated previously by Elliott et al. (2013) (Table 2).

**TABLE 2:** Summary of foraging metrics and their biological interpretation.

Breeding stage	Foraging metrics	Name	Biological interpretation
Incubation	Foraging principal component–maximum trip distance, average daily distance, mean trip distance, mean trip duration and number of trips per day	fPC1	Higher fPC1 scores indicate murres are making fewer trips, foraging at greater distances and durations with higher average daily distances
Chick rearing	Foraging principal component–maximum trip distance, mean trip distance, mean trip duration and number of trips per day	fPC1	Higher fPC1 scores indicate murres are making fewer trips and foraging at greater distances and durations
Chick rearing	Average daily distance (km)	dailyDist	Average distance traveled per day during foraging trips
Both	Average daily energy expenditure (kJ/day)	DEE	Average amount of energy expended per day based on daily acitivity budgets

We used a principal components analysis (PCA) to collapse down the multiple foraging variables we extracted from GPS units during both the incubation and chick‐rearing stages. The incubation PCA generated a single eigenvalue greater than one, explaining 65.3% of the variation, with maximum distance, average daily distance, mean trip distance, and mean trip duration strongly positively loaded onto factor one and number of trips per day strongly negatively loaded onto factor one (fPC1; Table 2, Table S1). The chick‐rearing PCA generated two eigenvalues greater than one, collectively explaining 86.4% of the variation, where maximum distance, mean trip distance, and mean trip duration were strongly positively loaded onto factor one, and number of trips per day was strongly negatively loaded onto factor one. Average daily distance weakly loaded onto factor one and was the only variable strongly loaded onto factor two. We therefore chose to remove this term from the PCA and test this variable separately. After removal of average daily distance from the PCA we had a single eigenvalue greater than one, explaining 75.7% of the variation, where maximum distance, mean trip distance, and mean trip duration were strongly positively loaded onto factor one and number of trips per day was strongly negatively loaded onto factor one (fPC1; Table 2, Table S2).

To visualize the foraging area of murres during breeding stages (for all study years) at the two colonies, we used kernel density analysis. We calculated the 95% and 50% utilization distributions from foraging locations (GPS locations categorized as on the water; excluding locations categorized as flying or at the colony) using the *adehabitatHR* package (Calenge, 2006).

### 2.5 Nutritional state and foraging success

We quantified nutritional biomarkers—plasma triglycerides (TRIG), baseline corticosterone (bCORT), beta‐hydroxybutyrate (B‐OH), and non‐esterified fatty acids (NEFA) in plasma samples to obtain pre‐foraging levels, post‐foraging levels, and relative change of nutritional biomarkers (∆ = log(post‐foraging levels) – log(pre‐foraging levels)) as estimates of nutritional state and foraging success (Table 1). To calculate coefficients of intra‐ and inter‐assay variation, we ran a control within and across sample assay plates for all study years (Table S3). We used a previously validated commercially available assay kit to measure plasma triglycerides (mmol/L; #TR0100‐1KT; Sigma Aldrich; Williams et al., 2007). We used a commercial enzyme‐linked immunoassay kit (EIA; Assay Designs) at a 1:40 dilution in triplicate to measure bCORT (ng/mL; Hennin et al., 2015). We used a previously‐validated kinetic assay to measure B‐OH (mmol/L; SIGMA, Guglielmo et al., 2002; Lamarre et al., 2017). We used a commercially available assay kit to measure NEFA (mmol/L; NEFA HR2, Wako Diagnostics; Smith et al., 2007; Jeanniard du Dot et al., 2009). For detailed methods on assays see Supplementary File.

### 2.6 Statistical analysis

#### 2.6.1 Environmental conditions

We used a Kruskall–Wallis test followed by a pairwise Wilcox‐test with a Bonferroni p‐value adjustment on daily mean sea ice concentration and sea surface temperature for each colony, to categorize years as high ice regime years (high sea ice concentration, cooler sea surface temperatures) or low ice regime years (low sea ice concentration, warmer sea surface temperatures).

#### 2.6.2 Intercolony and intracolony comparisons of foraging behavior, average daily energy expenditure, and foraging success

As foraging behavior is known to vary between breeding stages due to the demands of chick‐provisioning (Croll et al., 1991; Elliott, Woo, Gaston, Benvenuti, et al., 2009; Gaston & Hipfner, 2006a), we ran separate analyses for incubation and chick‐rearing stages for both intercolony comparisons and intracolony models. All variables were log‐transformed, with the exception of fPC1, body mass, and relative change in nutritional state, to meet normality assumptions. We fitted linear mixed models (LMMs) using *lme4* (Bates et al., 2015) with band number (individual ID) fitted as a random effect to account for repeated samples of individuals to compare GPS deployment duration, foraging behavior, average daily energy expenditure, pre‐foraging nutritional state, post‐foraging nutritional state, and foraging success (relative change in nutritional state) between colonies. When a low number of repeated individuals precluded the use of LMMs, we fit linear models, and repeated individuals were removed to meet test assumptions. Year was included in all models to account for interannual variation and GPS deployment duration was included as a fixed effect, when significant, to account for variation in deployment lengths. For nutritional state and foraging success models, time at the colony before the bird was sampled after returning from a foraging trip (TimebfSampling) was included as a fixed effect, if significant, to account for changes in physiological parameters over time.

To assess the impact of sea ice regime on foraging behavior, average daily energy expenditure, nutritional state, and foraging success of murres at both colonies during both breeding stages, we fitted LMMs, where band number (individual ID) and the start date of the deployment were fitted as random effects to account for repeated samples of individuals (when sample size was sufficient) and to account for temporal autocorrelation, respectively (see Supplementary File). When a low number of repeated individuals precluded the use of LMMs, we fit linear models, and repeated individuals were removed to meet test assumptions. For all models, sex and year (if there were more than two years) were fitted as fixed effects to account for sex differences and interannual variation, and GPS deployment duration was also included as a fixed effect, when significant, to account for variation in deployment lengths. For nutritional state and foraging success models, time at the colony before the bird was sampled after returning from a foraging trip (TimebfSampling) was included as a fixed effect, if significant, to account for changes in physiological parameters over time.

To ensure model assumptions were met for fixed and random effects, we visually inspected residuals versus fitted value plots to assess homogeneity of variance and quantile‐quantile plots to assess normality. We fitted full models using maximum likelihood estimation and used likelihood ratios tests (LRT) to test for significance of interactions between fixed effects, if interactions were nonsignificant (*p* > .05) they were removed from the model. We then re‐fit models using restricted maximum likelihood estimation (REML) and used *lmertest* (Kuznetsova et al., 2017) to obtain t‐statistics and *p*‐values. We calculated marginal *R* ^2^ ($r_{m}^{2}$; the proportion of variance in the model explained by the fixed effects) and conditional *R* ^2^ ($r_{c}^{2}$; the proportion of variance in the model explained by both fixed and random effects) for all models via *MuMIn* (Nakagawa et al., 2017; Barton 2022).

## 3 RESULTS

### 3.1 Intra‐Colony variation in environmental conditions

**FIGURE 1:**
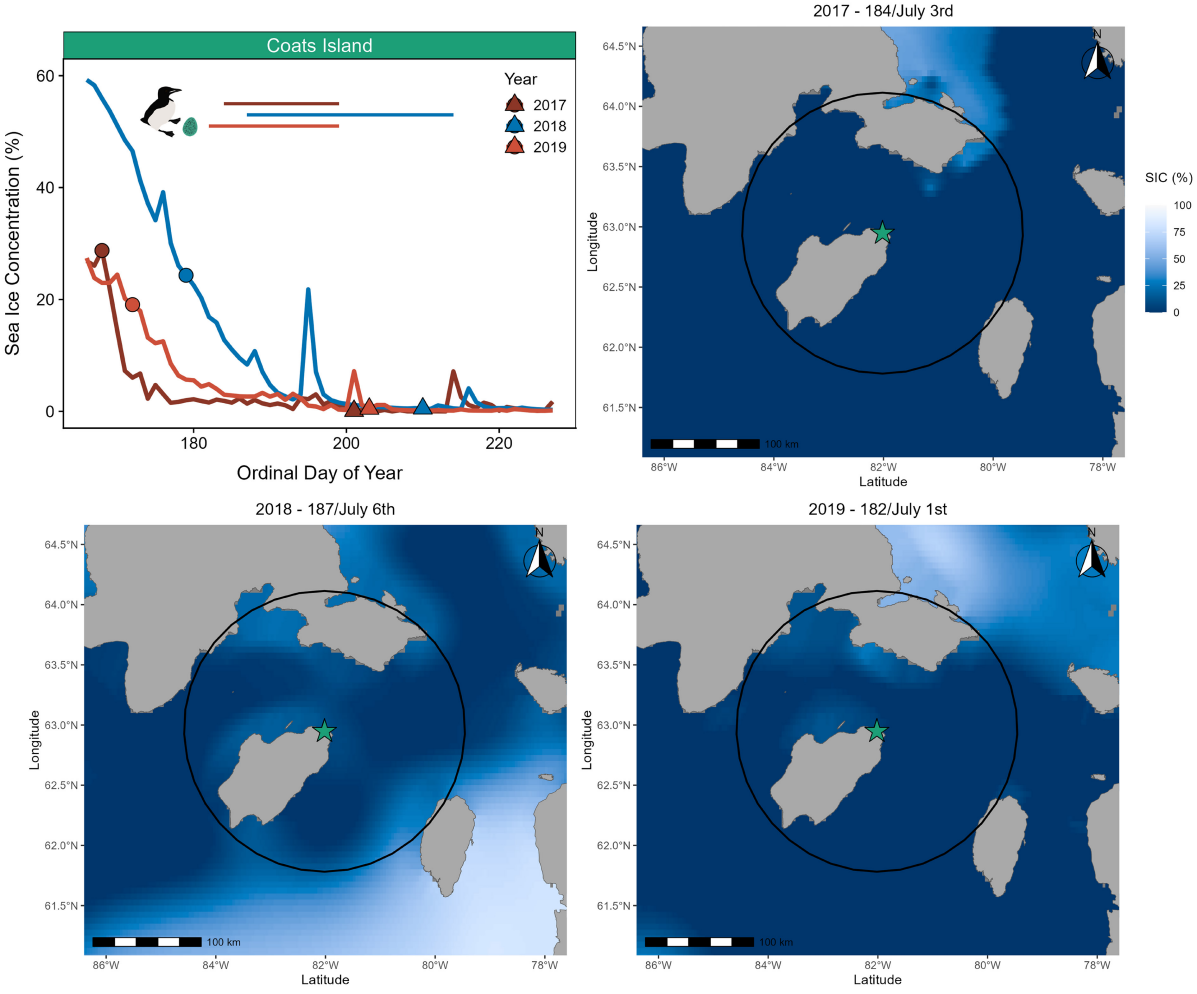
Sea ice concentration (%) throughout the thick‐billed murre breeding period (15 June to 15 August) at Coats Island, Nunavut (top left panel), circles depict mean lay dates and triangles depict mean hatch dates respective to study years, straight horizontal lines indicate the incubation GPS deployment range for each study year. Low ice regime years (low sea ice concentration, high sea surface temperarture) are shown in red and light red (2017 and 2019) and high ice regime years (high sea ice concentration, low sea surface temperature) are shown in blue (2018). Maps show sea ice concentration (SIC; %) on the first day of GPS deployments in 2017 (ordinal day of year 184; July 3rd; top right panel), 2018 (ordinal day of year 187; July 6th; bottom left panel), and 2019 (ordinal day of year 182; July 1st; bottom right panel), black circle indicates the maximum foraging range (130 km) of thick‐billed murres at Coats Island (turquoise star).

**FIGURE 2:**
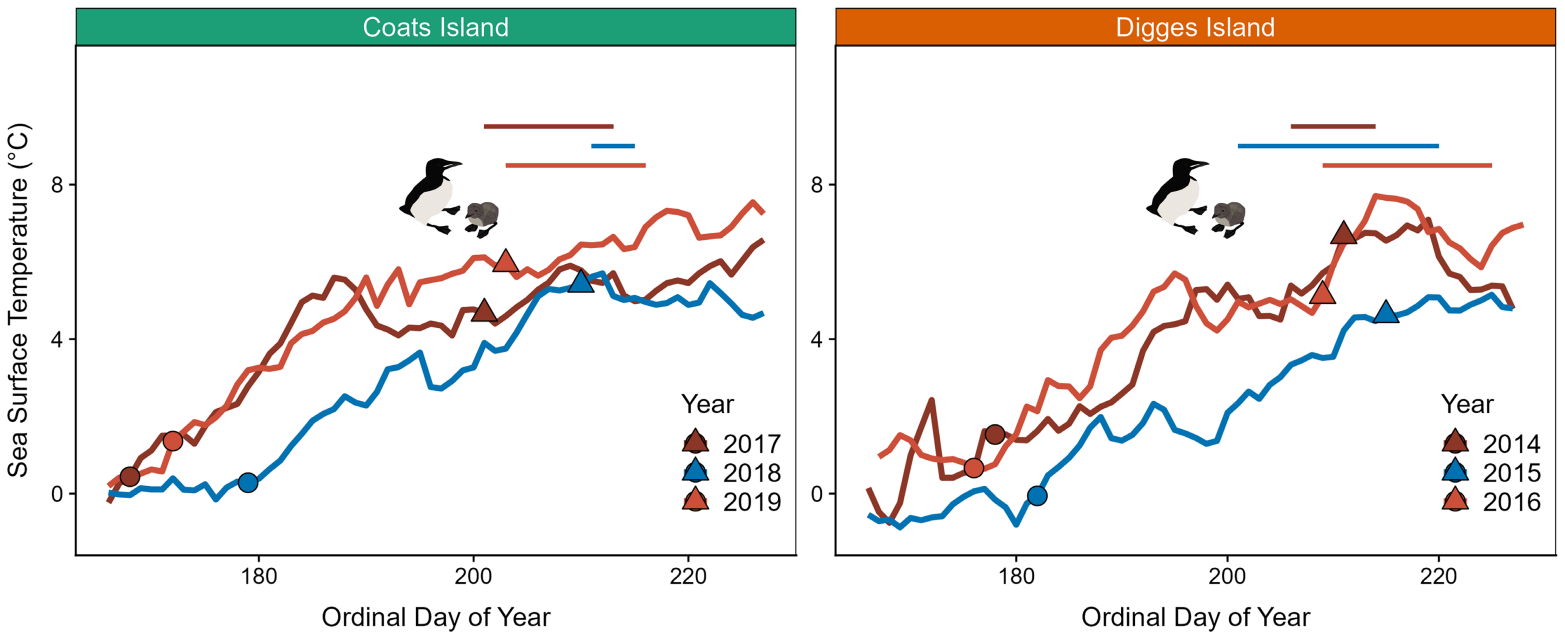
Sea surface temperature (°C) throughout the thick‐billed murre breeding period (15 June to 15 August) at Coats Island, Nunavut (left panel) and Digges Island, Nunavut (right panel), circles depict mean lay dates and triangles depict mean hatch dates respective to study years, straight horizontal lines indicate the chick‐rearing GPS deployment range for each study year. Low ice regime years (low sea ice concentration, high sea surface temperarture) are shown in red and light red (2014, 2016, 2017, and 2019), and high ice regime years (high sea ice concentration, low sea surface temperature) are shown in blue (2015 and 2018).

**FIGURE 3:**
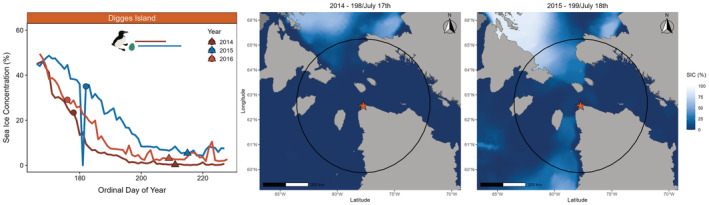
Sea ice concentration (%) throughout the thick‐billed murre breeding period (15 June to 15 August) at Digges Island, Nunavut (left panel), circles depict mean lay dates and triangles depict mean hatch dates respective to study years, straight horizontal lines indicate the incubation GPS deployment range for each study year. Low ice regime years (low sea ice concentration, high sea surface temperarture) are shown in red and light red (2014 and 2016) and high ice regime years (high sea ice concentration, low sea surface temperature) are shown in blue (2015). Maps show sea ice concentration (SIC; %) on the first day of GPS deployments in 2014 (ordinal day of year 198; July 17th; center panel) and 2015 (ordinal day of year 199; July 18th; right panel), and black circle indicates the maximum foraging range (300 km) of thick‐billed murres at Digges Island (orange star).

Both sea ice concentration and sea surface temperature throughout the breeding season differed among years at both the Coats Island (sea ice concentration: H = 12.8, df = 2, *p* = .002; sea surface temperature: H = 31.3, df = 2, *p* < .001) and Digges Island colonies (sea ice concentration: *H* = 36.1, df = 2, *p* < .001; sea surface temperature: *H* = 28.7, df = 2, *p* < .001). For sea ice concentration at Coats Island, 2018 differed from both 2017 (*p*‐adjusted = .01) and 2019 (*p*‐adjusted = .01), whereas 2017 and 2019 did not differ (*p*‐adjusted = 1.0). For sea surface temperature at Coats Island, all years differed from each other (2017–2018 *p*‐adjusted = .001; 2017–2019 *p*‐adjusted = .02; 2018–2019 *p*‐adjusted <.001). We therefore categorized 2019 and 2017 as warm, low ice years (low ice regime; Figures 1 and 2; Figure S1) and 2018 as a cool, high ice year (high ice regime; Figures 1 and 2; Figure S1). For sea ice concentration at Digges Island, all years differed from each other (2014–2015 *p*‐adjusted <.001; 2014–2016 *p*‐adjusted = .001; 2015–2016 p‐adjusted = 0.001). For sea surface temperature at Digges Island, 2015 differed from both 2014 (*p*‐adjusted <.001) and 2016 (*p*‐adjusted <.001), whereas 2014 and 2016 did not differ (p‐adjusted = 0.78). We therefore categorized 2014 and 2016 as warm, low ice years (low ice regime; Figures 2 and 3; Figure S2) and 2015 as a cool, high ice regime year (high ice regime; Figures 2 and 3; Figure S2). Although there was no overlap in study years between sites, environmental conditions (sea ice concentration and sea surface temperature) followed the same trends during the breeding season at both sites. Futhermore, both sites experienced two low ice regime years and one high ice regime year, allowing us to make intercolony comparisons of foraging behavior, average daily energy expenditure, pre‐ and post‐foraging nutritional state, and foraging success.

### 3.2 Intercolony comparisons of foraging behavior, energy expenditure, and nutritional biomarkers

**FIGURE 4:**
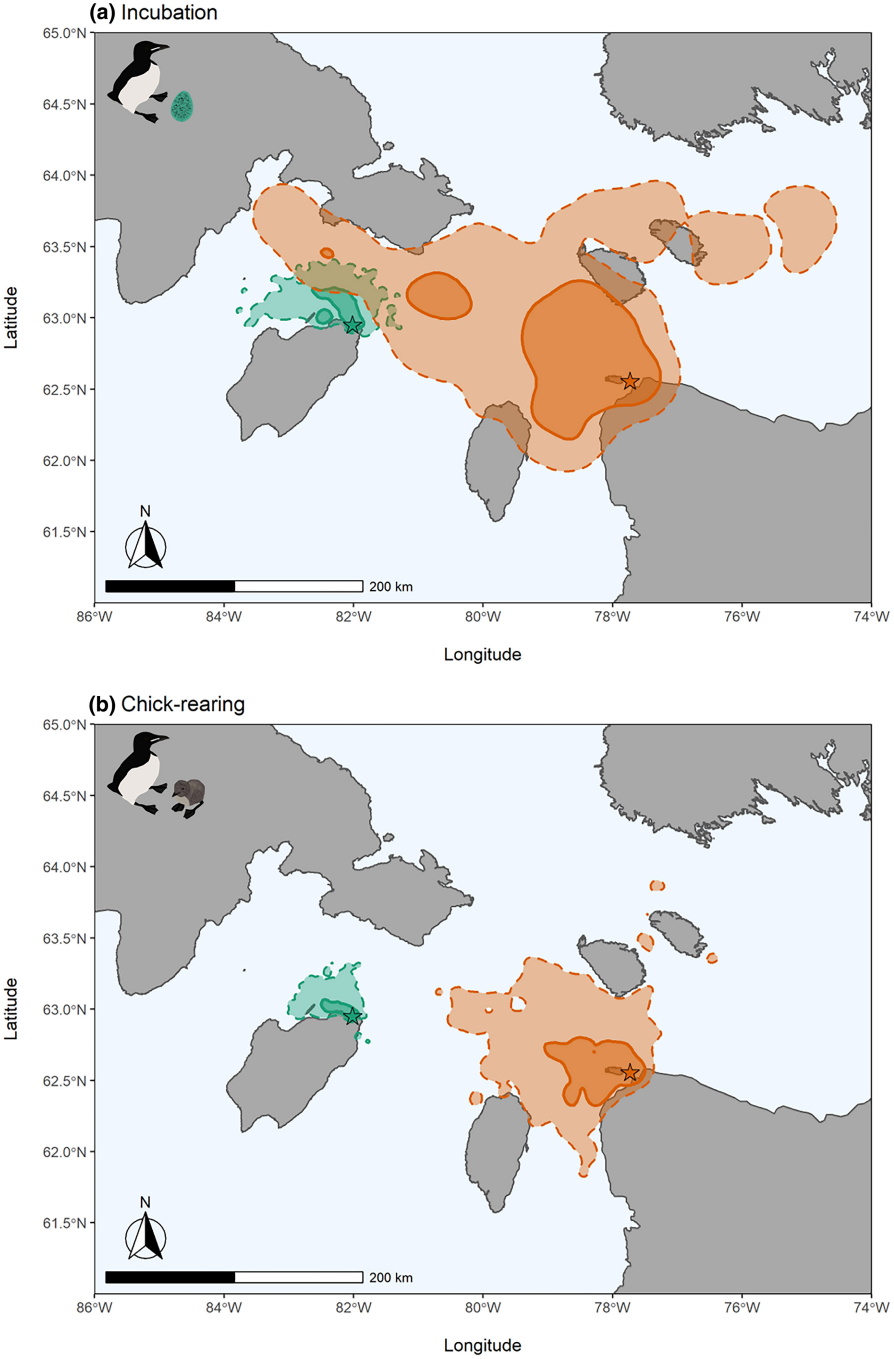
Foraging distribution of thick‐billed murres from Coats Island, Nunavut (turquoise; 2017, 2018, and 2019) and Digges Island, Nunavut (orange; 2014, 2015, and 2016) during incubation (top panel) and chick‐rearing (bottom panel) stages. Dashed lines represent the overall foraging area (95% utilization distributions), and solid lines represent the core foraging area (50% utilization distributions). Stars represent colony locations.

**FIGURE 5:**
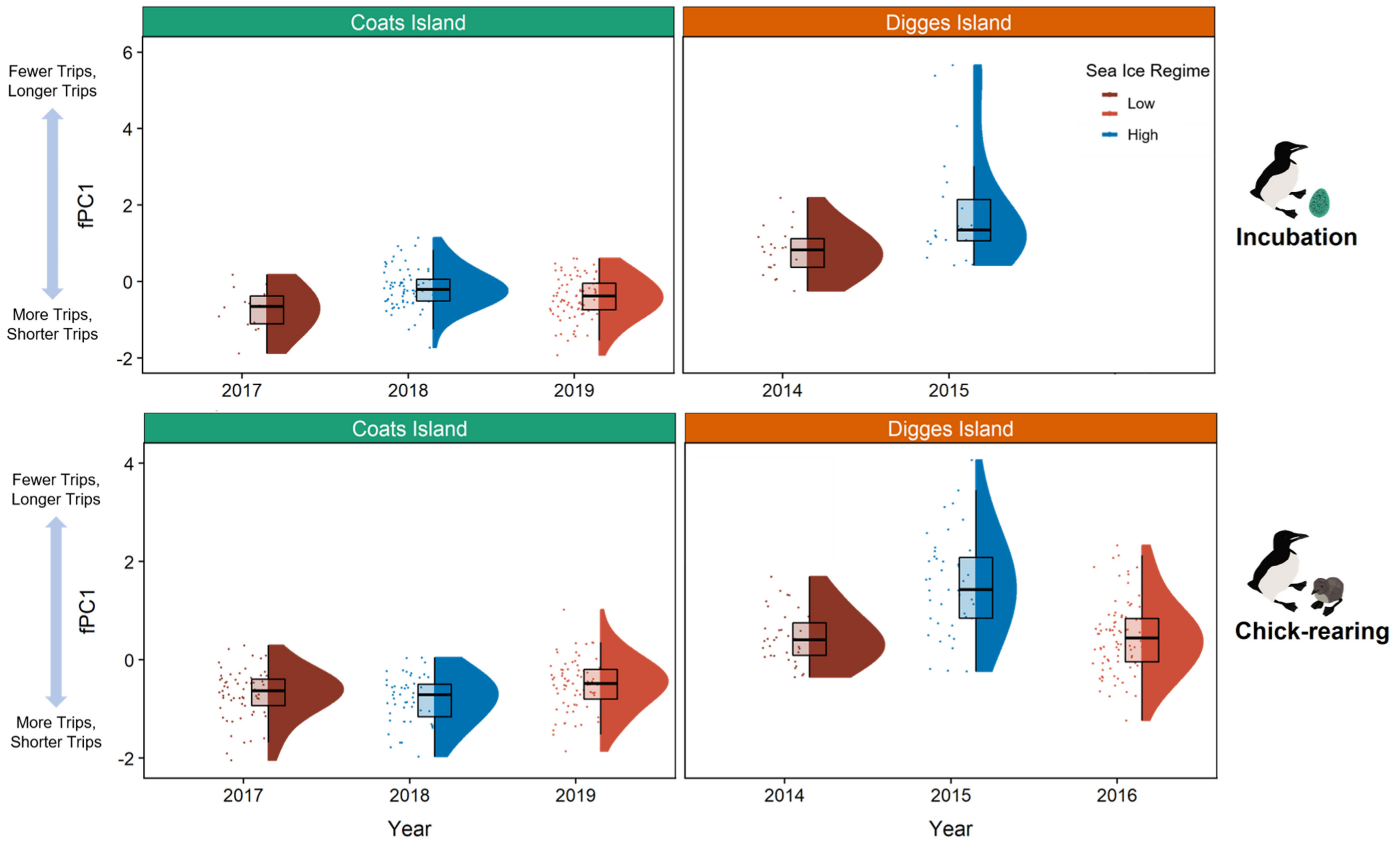
Intercolony variation in thick‐billed murre foraging behavior during the incubation stage (fPC1—maximum distance traveled, average daily distance, mean trip distance, mean trip duration, and number of trips per day; top panels) and chick‐rearing stage (fPC1—maximum distance traveled, mean trip distance, mean trip duration, and number of trips per day; bottom panels) at Coats Island, Nunavut (left panels; turquoise) in 2017 (dark red; low sea ice regime), 2018 (blue; high sea ice regime), and 2019 (light red; low sea ice regime), and at Digges Island, Nunavut (right panels; orange) in 2014 (dark red; low sea ice regime), 2015 (blue; high sea ice regime), amd 2016 (light red; low ice regime).

Several foraging and nutritional biomarkers differed between colonies during both incubation and chick rearing (Table 3; Tables S4–S12). During incubation, an interaction between colony and GPS deployment duration revealed that murres making fewer, longer trips (higher fPC1 scores) have longer GPS deployments at Coats but not at Digges (*p* = .03, Table S4). Overall, murres at the larger Digges Island colony made fewer trips, and trips were longer in duration and distance with higher average daily distances (higher fPC1 scores; Table 3, Table S4, Figures 4 and 5) relative to murres at Coats Island. Similarly, an interaction between colony and duration on average daily energy expenditure revealed that at Coats average daily energy expenditure increases with GPS deployment duration, where as, the opposite is observed at Digges (*p* = .001, Table S4), where overall incubating murres at Digges Island had higher average daily energy expenditure (Table 3, Table S4). Additionally, incubating murres at Digges had a lower nutritional state (lower pre‐ and post‐foraging mass, higher pre‐ and post‐foraging bCORT, higher pre‐foraging B‐OH, and higher pre‐ and post‐foraging NEFA; Table 3, Tables S5–S7). An interaction between colony and duration on post‐foraging TRIG (*p* = .03, Table S6) and relative change in TRIG (*p* = .002, Table S8) revealed that nutritional state and foraging success declined with GPS deployment duration at Coats, and overall, nutritional state and foraging success was lower at Digges: lower post‐foraging TRIG (Table 3, Table S6) and relative change in TRIG (Table 3, Table S8).

**TABLE 3:** Intercolony comparisons (mean ± standard error—SE; log‐scaled when necessary) of GPS deployment duration, foraging behavior (incubation: fPC1—maximum distance, average daily distance, mean trip distance, mean trip duration, and number of trips per day; chick rearing: fPC1—maximum distance, mean trip distance, mean trip duration, and number of trips per day and dailyDist—average daily distance), average daily energy expenditure (DEE), and pre‐foraging, post‐foraging, and relative change in nutritional state (mass, baseline corticosterone—bCORT, non‐esterified fatty acids—NEFA, beta‐hydroxybutyrate—B‐OH, and triglycerides—TRIG) during the incubation and chick‐rearing stages at Coats Island, Nunavut (turquoise) and Digges Island, Nunavut (orange). Statistical comparisons (purple) were made using linear mixed models and linear models with significant *p*‐values shown in bold.

	Variable	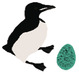 Incubation							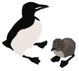 Chick rearing						
		Coats Island		Digges Island		Statistics			Coats Island		Digges Island		Statistics		
		Mean + SE	*n*	Mean + SE	*n*	df	*t*	*p*	Mean + SE	*n*	Mean + SE	*n*	df	*t*	*p*
GPS deployment	log Duration	0.86 ± 0.02	170	1.70 ± 0.07	44	202	11.5	**<.001**	0.66 ± 0.02	186	1.25 ± 0.04	162	341	9.18	**<.001**
Foraging behavior	fPC1	−0.34 ± 0.04	170	1.31 ± 0.19	44	207	4.47	**<.001**	−0.65 ± 0.04	186	0.74 ± 0.07	162	336	5.18	**<.001**
	dailyDist (km)	—	—	—	—	—	—	—	154.7 ± 4.08	186	142.4 ± 3.36	162	342	0.01	.99
Avgerage daily	log DEE	7.65 ± 0.01	170	7.84 ± 0.03	44	207	5.20	**<.001**	—	—	—	—	—	—	—
Energy expenditure	DEE (kJ/day)	—	—	—	—	—	—	—	2566.3 ± 36.7	186	2616.5 ± 36.3	145	337	1.67	.10
Pre‐foraging nutritional state	Mass (g)	1032 ± 5	162	995 ± 9	42	199	−1.95	**.05**	975 ± 4	186	959 ± 5	156	333	−1.10	.27
	log bCORT	0.33 ± 0.08	131	1.80 ± 0.12	40	166	7.42	**<.001**	0.88 ± 0.10	70	1.68 ± 0.09	87	190	6.09	**<.001**
	log NEFA	−1.46 ± 0.05	131	−0.55 ± 0.07	39	121	7.51	**<.001**	−1.00 ± 0.05	71	−0.63 ± 0.04	121	187	6.07	**<.001**
	log B‐OH	−0.05 ± 0.03	131	0.06 ± 0.08	37	139	2.38	**.02**	0.40 ± 0.03	71	0.39 ± 0.04	118	183	−1.15	.25
	log TRIG	−0.20 ± 0.04	124	−0.30 ± 0.08	34	—	−1.59	.11	0.16 ± 0.06	71	−0.15 ± 0.04	124	190	−3.89	**<.001**
Post‐foraging nutritional state	Mass (g)	994 ± 5	158	943 ± 12	27	153	−2.44	**.02**	951 ± 4	182	917 ± 6	105	280	−3.46	**.001**
	log bCORT	0.80 ± 0.09	125	1.82 ± 0.17	24	—	4.42	**<.001**	0.88 ± 0.10	70	1.68 ± 0.09	87	152	5.31	**<.001**
	log NEFA	−1.32 ± 0.05	125	−0.37 ± 0.09	24	—	6.56	**<.001**	−1.00 ± 0.07	70	−0.45 ± 0.06	87	153	6.37	**<.001**
	log B‐OH	0.12 ± 0.03	132	0.22 ± 0.11	27	155	−1.40	.17	0.44 ± 0.04	70	0.48 ± 0.05	82	—	0.08	.94
	log TRIG	−0.35 ± 0.04	132	−0.52 ± 0.12	26	150	−3.68	**<.001**	0.02 ± 0.07	65	−0.25 ± 0.05	82	—	−0.39	.70
Relative change in nutritional state	Mass	−0.04 ± 0.00	150	−0.05 ± 0.01	23	—	−2.09	**.04**	−0.03 ± 0.00	182	−0.05 ± 0.01	103	278	−4.28	**<.001**
	bCORT	0.48 ± 0.11	124	0.32 ± 0.20	22	—	−0.46	.65	−0.00 ± 0.13	70	0.33 ± 0.11	84	150	1.77	.08
	NEFA	0.12 ± 0.07	124	0.17 ± 0.13	22	—	−0.45	.66	−0.01 ± 0.08	65	0.21 ± 0.07	81	—	1.55	.12
	B‐OH	0.17 ± 0.04	131	0.27 ± 0.11	24	151	−1.80	.07	0.05 ± 0.05	70	0.09 ± 0.07	76	—	0.53	.60
	TRIG	−0.15 ± 0.04	124	−0.30 ± 0.14	22	—	−3.24	**.001**	−0.12 ± 0.06	65	−0.11 ± 0.06	80	—	0.8	.46

Chick‐rearing murres at the larger Digges Island made fewer trips, and trips were longer in duration and distance (higher fPC1 scores; Table 3, Table S9, Figures 4 and 5), relative to murres at Coats Island. While average daily distance and average daily energy expenditure did not differ between colonies during the chick‐rearing stage (Table 3, Table S9), murres at Digges had a lower nutritional state (lower post‐foraging mass, lower pre‐foraging TRIG, higher pre‐ and post‐foraging bCORT, and higher pre‐ and post‐foraging NEFA Table 3, Tables S10 and S11) and lower foraging success (lower relative change in mass; Table 3, Table S11).

### 3.3 Intracolony comparisons of foraging behavior, energy expenditure, and foraging success

#### 3.3.1 Digges Island (larger colony)

##### Incubation

Murres made more trips, and trips were shorter in duration and distance with lower average daily distances in low ice regimes (lower fPC1 scores; *p* < .01; Table 4, Table S13, Figure 5). Likewise, sea ice regime interacted with foraging behavior to influence average daily energy expenditure (*p* = .01; Table 4, Table S14), where murres had higher average daily energy expenditure when making fewer trips that were longer in duration and distance with greater average daily distances, with the slope of this relationship being steeper under low ice regimes. Under low ice regimes murres had a higher nutritional state and higher foraging success: lower post‐foraging B‐OH (*p* = .002; Table 4, Table S15) and lower relative change in B‐OH (*p* = .03; Table 4, Table S15).

**TABLE 4:** Summary of output from linear mixed models and linear models for foraging behavior (incubation: fPC1—maximum trip distance, average daily distance, mean trip distance, mean trip duration, and number of trips per day; chick rearing: average daily distance traveled and fPC1—maximum trip distance, mean trip distance, mean trip duration, and number of trips per day), average daily energy expenditure (DEE), post‐foraging levels (post‐) and relative change (∆) of nutritional biomarkers (mass, baseline corticosterone—bCORT, nonesterified fatty acids—NEFA, beta‐hydroxybutyrate—B‐OH, and triglycerides—TRIG) at Digges Island, Nunavut (orange shading) and Coats Island, Nunavut (turquoise shading) during the incubation and chick‐rearing breeding stages. Arrows and shading depict direction of change when there is a significant difference, an upwards arrow (↑) and darker shading represents an increase and a downwards arrow (↓) and lighter shading represents a decrease, a yellow star (*) depicts a significant interaction between variables, a white equal sign (=) depicts no significant difference, and a gray dash (—) represents no data.

		Digges Island		Coats Island	
		Sea Ice Regime		Sea Ice Regime	
Breeding stage	Variable	Low	High	Low	High
Incubation	fPC1	↓	↑	↓	↑
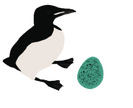	DEE	*	*	*	*
	post‐Mass	=	=	↓	↑
	∆ Mass	=	=	↓	↑
	post‐bCORT	=	=	=	=
	∆ bCORT	=	=	=	=
	post‐NEFA	=	=	=	=
	∆ NEFA	=	=	=	=
	post‐B‐OH	↓	↑	=	=
	∆ BOH	↓	↑	=	=
	post‐TRIG	=	=	*	*
	∆ TRIG	=	=	=	=
Chick rearing	fPC1	↓	↑	=	=
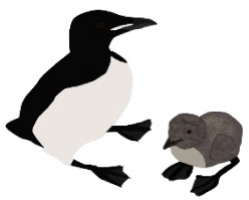	dailyDist	↑	↓	=	=
	DEE	*	*	↑	↓
	post‐Mass	=	=	↓	↑
	∆ Mass	*	*	=	=
	post‐bCORT	*	*	—	—
	∆ bCORT	=	=	—	—
	post‐NEFA	↑	↓	—	—
	∆ NEFA	=	=	—	—
	post‐B‐OH	=	=	—	—
	∆ BOH	=	=	—	—
	post‐TRIG	=	=	—	—
	∆ TRIG	=	=	—	—

##### Chick rearing

Similar to incubation, chick‐rearing murres made more trips of shorter duration and distance (lower fPC1 scores; *p* < .001; Table 4, Table S16, Figure 5) and had higher average daily distances (*p* = .01; Table 4, Table S16) in low ice regimes. Additionally, murres had higher average daily distances when making fewer trips of longer duration and distance (*p* < .001; Table S16). Sea ice regime interacted with fPC1 to influence average daily energy expenditure (*p* = .02; Table 4, Table S17), where murres had slightly lower average daily energy expenditure when making more trips of shorter duration and distance in low ice regimes, whereas in high ice regimes, murres had lower average daily energy expenditure when making fewer trips of longer duration and distance. Sea ice regime also interacted with average daily distance to influence average daily energy expenditure (*p* = .01; Table 4, Table S17), where murres had higher average daily energy expenditure when average daily distance traveled was highest, with the slope of this relationship being steeper in a high ice regime. In addition, murres had a lower nutritional state in low ice regimes: higher post‐foraging NEFA (*p* = .01; Table 4, Table S16). An interaction between sea ice regime and GPS deployment duration also revealed that foraging success (relative change in mass) declined with longer GPS deployments during low ice regimes, whereas in high ice regimes foraging success remained stable across deployment lengths (*p* = .03, Table S17). Sea ice regime also interacted with average daily distance to influence post‐foraging bCORT (*p* = .04; Table 4, Table S16), where murres had lower post‐foraging bCORT when traveling lower average daily distances in low ice regimes, the opposite occurred in high ice regimes.

### 3.4 Coats Island (smaller colony)

#### 3.4.1 Incubation

Similar to the larger Digges Island colony, murres made more trips of shorter duration and distance with lower average daily distances in low ice regimes (lower fPC1 scores; *p* = .001; Table 4, Figure 5, Table S19). An interaction between sea ice and fPC1 on average daily energy expenditure (*p* = .001; Table 4, Table S19) revealed that while average daily energy expenditure generally increased as murres made fewer trips of longer duration and distance with higher average daily distances, the slope of this relationship was higher during a high ice regime. Additionally, nutritional state and foraging success were lower in low ice regimes: lower post‐foraging mass (*p* < .001; Table 4; Table S20); and lower relative change in mass (*p* = .002; Table 4; Table S20). Lastly, an interaction between sea ice regime and GPS deployment duration revealed that post‐foraging TRIG declined more steeply with longer deployment durations under low ice regimes (*p* = .03; Table 4; Table S19).

#### 3.4.2 Chick rearing

Unlike during the incubation stage, sea ice regime did not influence foraging behavior—fPC1 (*p* = .23; Table 4, Table S22, Figure 5) or average daily distance traveled (*p* = .57; Table 4, Table S22). Regardless, average daily energy expenditure was higher in low ice regimes (*p* = .05; Table 4, Table S22), with average daily energy expenditure increasing with average daily distance traveled (*p* < .001; Table 4, Table S22). Similar to incubation, murres had a lower nutritional state in low ice regimes (lower post‐foraging mass, *p* = .01; Table 4, Table S23).

## 4 DISCUSSION

Our multiyear, integrative field study revealed some of the intrinsic and extrinsic drivers of inter‐ and intracolony variation in foraging behavior and foraging success in an Arctic‐breeding seabird facing rapid environmental change. The northern Hudson Bay region where our study took place has seen a consistent decline in summer sea ice extent over the past thirty years (Gaston & Elliott, 2014). We first confirmed there is interannual variation in sea ice concentration and sea surface temperature at both colonies. Although study years did not overlap between colonies, birds experienced similar environmental conditions during the breeding seasons, with both colonies experiencing high and low ice regimes during the study period, allowing for intercolony comparisons to be made. Second, consistent with the prediction that larger colonies should deplete resources surrounding the colony quicker than smaller colonies (Ashmole, 1963), we found that murres at the larger colony foraged farther, resulting in lower foraging success. Third, we observed behavioral flexibility in response to environmental change at both colonies during incubation, where murres made fewer and more distant foraging trips in high ice regimes. The same trend was observed during chick rearing, but only at the larger colony. Although murres at the smaller colony did not exhibit foraging flexibility during chick rearing, foraging success at both colonies was higher (higher post‐foraging mass at Coats and lower post‐foraging NEFA at Digges) in high ice regimes, suggesting greater prey abundance and availability. Taken together, we expect that larger Arctic seabird colonies will be more sensitive to climate change.

### 4.1 Intercolony variation in foraging behavior, energy expenditure, and foraging success

During incubation, murres are only constrained by their partner's ability to remain at the nest; therefore, foraging trips are longer during this time, allowing murres to exploit more distant prey patches (Croll et al., 1991). Murres at the larger colony traveled further and made longer but fewer trips with higher average daily distances compared to the smaller colony (Figure 4). This trend is consistent with Ashmole's halo hypothesis (Ashmole, 1963) and supported empirically in murres (Elliott, Woo, Gaston, Benvenuti, et al., 2009; Gaston et al., 2007; Patterson et al., 2022), where larger colonies have larger foraging ranges, likely resulting from depleted resources around the colony. Furthermore, these same birds at the larger colony also had a lower nutritional state and lower foraging success compared to murres at a smaller colony. Differences in nutritional state and foraging success between colonies were likely a result of contrasting foraging behavior, suggesting higher energetic costs associated with more distant foraging at a large colony (Elliott et al., 2013).

Similarly, during the chick‐rearing stage, murres at the larger colony had a lower nutritional state and lower foraging success. Although foraging behavior (foraging principal component, fPC1) still varied between colonies during chick rearing, with murres at the larger colony making fewer trips and foraging farther from the colony, average daily distance traveled and average daily energy expenditure of murres did not vary between colonies. This suggests that birds at both colonies respond to the increased pressures of chick demand (while still fueling somatic needs) with different foraging strategies that nonetheless optimize mean distance traveled per day. However, the lower foraging success seen in murres at the larger colony, Digges Island, likely reflects higher interspecific competition for prey during chick rearing and negative carry‐over effects from lower payoffs during the incubation stage (lower prey availability surrounding the colony as a function of density dependence) (Gaston & Hipfner, 2006b; Hipfner et al., 2006).

Interestingly, pre‐foraging mass did not vary between colonies during the chick‐rearing period. Murres lose mass in late incubation to reduce wing‐loading during the energetically expensive chick‐rearing stage (Croll et al., 1991), and our results suggest murres reduced mass to their absolute minimum threshold (Gaston & Hipfner, 2006a). However, the higher post‐foraging mass and relative change of mass of murres at the smaller colony during chick rearing likely reflect greater energetic payoffs due to higher prey availability and abundance surrounding the colony, as a result of reduced intraspecific competition due to smaller colony size (Gaston & Hipfner, 2006a, 2006b; Hipfner et al., 2006). Similarly, previous findings comparing murres from Coats and Digges Island found that during chick‐rearing murres at the larger Digges Island had lower mass and lower chick‐growth rates (Gaston & Hipfner, 2006b; Hipfner et al., 2006).

### 4.2 Intracolony variation in foraging behavior, energy expenditure, and foraging success

#### 4.2.1 Larger colony—Digges Island

Murres at the larger colony made fewer but longer trips under high ice regimes across both reproductive stages. However, average daily distance was higher under low ice regimes during chick rearing, which could represent increased search time for prey. During incubation, we observed lower nutritional state and foraging success (higher levels and greater relative change of beta‐hydroxybutyrate) during high ice regimes, initially suggesting lower prey availability. However, this could reflect high flight costs associated with more distant foraging trips (Elliott et al., 2013). Alternatively, higher levels of beta‐hydroxybutyrate could result from longer fasting periods at the nest given longer foraging trips by their partner during high ice regimes. Longer flights could represent a high energy search strategy (Norberg, 2021), suggesting murres could be tracking distant ice edges, cueing into foraging locations with higher prey availability when ice is still present. For example, in Iceland, thick‐billed murres are cold‐water specialists, selectively foraging in cooler waters in fjords and along the Marginal Ice Zone (Bonnet‐Lebrun, Larsen, Frederiksen, et al., 2021; Bonnet‐Lebrun, Larsen, Thórarinsson, et al., 2021). As we then observed a higher nutritional state (lower levels of non‐esterified fatty acids) within a high ice regime during the chick‐rearing stage, this further suggests prey availability may have been higher during a high ice regime.

Furthermore, as foraging success (relative change in mass) during chick rearing was only impacted by GPS deployment duration during low ice regimes, this also suggests that there was lower prey availability in low ice regimes. Interestingly, during chick rearing, when sea ice concentration was below 10% within the local foraging range of murres at the larger colony, murres had a higher nutritional state (lower post‐foraging baseline corticosterone) in low ice regimes when average daily distance traveled was low. The opposite was observed under high ice regimes, when murres had a higher nutritional state (lower post‐foraging baseline corticosterone) when average daily distance traveled was high. This suggests murres could be foraging on different prey items under different ice regimes, where one strategy is successful during high ice regimes, and a different strategy is successful under low ice regimes. As previously mentioned, under high ice regimes (when ice is still present during the breeding season), murres may be foraging on distant sympagic arctic cod, and murres exhibiting this foraging strategy were most successful, as baseline corticosterone was lowest when average daily distance was highest. Under low ice regimes, baseline corticosterone was lowest when murres had lower average daily distances traveled, suggesting murres that foraged for coastal prey were more successful. Notably, the proportion of sub‐arctic Atlantic fishes, e.g., capelin and Atlantic herring (*Clupea harengus*), has increased in the diet of black‐legged kittiwakes (*Rissa tridactyla*) breeding in Svalbard, Norway, with Atlantic fishes being more abundant in years with a lower sea ice index (Vihtakari et al., 2018). Previously collected stable isotope data and stomach content analysis from Digges Island reflected a high reliance on capelin and sandlance (Provencher et al., 2013), therefore capelin and sandlance could be more positively responding to warmer conditions leading to increased availability in low ice regimes (Vihtakari et al., 2018). If nutritional state associated with different foraging strategies (e.g., individuals specializing on specific prey, Elliott, Woo & Gaston, 2009; Provencher et al., 2013; Woo et al., 2008) depends on environmental conditions, it is possible that the presence of different foraging strategies could allow populations to buffer effects of environmental variability (Elliott et al., 2010).

#### 4.2.2 Smaller colony—Coats Island

During the incubation stage, under low ice regimes murres made more trips and shorter trips with lower average daily distances, compared to high ice regime years, when murres made fewer trips, foraging at further distances with higher average daily distances. While high ice concentration (>95%) has been shown to impede foraging of murres breeding at high Arctic sites, e.g., Prince Leopold Island (Gaston, Gilchrist, & Hipfner, 2005; Gaston, Gilchrist, & Mallory, 2005), sea ice concentration in our study was below 10% during GPS deployments suggesting that ice was likely not a constraining factor (i.e., ice was not physically impeding foraging at closer distances). Either strategy could then represent increased search time for prey, however, as we observed a higher nutritional state and higher foraging success (higher post‐foraging mass and greater relative change in mass) during a high ice regime this suggests prey availability and abundance was higher under high ice regimes. Additionally, under low ice regimes, the nutritional state of murres (post‐foraging triglycerides) declined more steeply with longer GPS deployments, further suggesting lower prey availability in low ice regimes.

These findings pose the question why murres would forage at greater distances if flight costs are high? As optimal foraging theory predicts that individuals will maximize energetic gain while minimizing energetic cost (MacArthur & Pianka, 1966; Pyke et al., 1977), it is therefore possible murres were foraging at distant hotspots, adjacent to ice edges with high concentrations of sympagic Arctic cod (Christensen‐Dalsgaard et al., 2017; LeBlanc et al., 2019). Previous work combining at‐sea seabird surveys with sea ice concentration and acoustic surveys found thick‐billed murres were observed in proximity to Arctic cod, that were ranging from 12 to 200 m in depth, where Arctic cod in that depth range were most frequently associated with 40%–60% ice cover (LeBlanc et al., 2019). Furthermore, previous biologging at Coats Island found murres typically catch Arctic cod around 60 metres in depth and require less energy to capture underwater (shortest dive bottom times) compared to other prey items, suggesting there is a trade‐off between flight costs and dive costs associated with Arctic cod (Elliott et al., 2008).

In contrast to the incubation stage, murres did not adjust foraging behavior (foraging principal component or average daily distance traveled) in response to sea ice regime during the chick‐rearing stage, likely reflecting the high cost of chick provisioning, constraining foraging closer to the colony. Regardless of environmental conditions, lower intraspecific competition surrounding a small colony may leave adequate prey resources during the chick‐rearing stage to allow for closer foraging. Furthermore, as adults must maximize foraging for chicks, they may switch to more benthic or invertebrate prey that is found at closer distances (Brisson‐Curadeau & Elliott, 2019; Gaston & Elliott, 2014). The absence of sea ice within the foraging range of murres at Coats Island during the chick‐rearing stage across sea ice regimes may have also lead to a greater reliance on less distant benthic or invertebrate prey (Brisson‐Curadeau & Elliott, 2019; Gaston & Elliott, 2014). Unfortunately, due to a reduced dataset during chick rearing we could not make broad‐scale environmental comparisons of foraging success from nutritional biomarkers (energetic hormones and metabolites) at the smaller colony. However, similar to incubation, nutritional state (post‐foraging mass) was also higher under a high ice regime during the chick‐rearing stage, again suggesting greater prey availability.

## 5 CONCLUSIONS

We used an integrative approach that combined behavior, energetics, and physiology to examine how environmentally mediated changes in foraging strategies ultimately impacted foraging success at different colony scales. Overall, our results suggest that larger colonies may be more susceptible to increasing Arctic change, through complex linkages between environmental variability and prey availability, ultimately impacting foraging flexibility and success during two key breeding stages. Though individuals from the two colonies experienced similar environmental conditions in the nonbreeding season (shared wintering grounds; Frederiksen et al., 2016) and breeding season (only 220 km apart), the larger colony was more sensitive to environmental variation during the breeding season. The degree of inter‐annual flexibility in foraging behavior seen in this study suggests murres as a species may have the behavioral flexibility to cope with current rates of climate change occurring in the Canadian Arctic. Nonetheless, because murres at a large colony had more drastic responses to environmental change and overall lower foraging success suggests these colonies may still have greater demographic sensitivity to environmental change. To further our understanding of the fitness and therefore population demography outcomes of these complex relationships, future studies should integrate measures of foraging and diving behavior and prey availability and abundance with breeding success and survival to ultimately determine how murres are affected by a changing climate.

## AUTHOR CONTRIBUTIONS

**Alyssa Eby:** Conceptualization (equal); data curation (equal); formal analysis (lead); funding acquisition (supporting); investigation (equal); methodology (equal); visualization (lead); writing – original draft (lead); writing – review and editing (equal). **Allison Patterson:** Data curation (equal); formal analysis (supporting); methodology (supporting); writing – review and editing (equal). **Graham Sorenson:** Conceptualization (equal); data curation (equal); funding acquisition (supporting); investigation (equal); writing – review and editing (equal). **Thomas Lazarus:** Data curation (equal); writing – review and editing (equal). **Shannon Whelan:** Data curation (equal); formal analysis (supporting); writing – review and editing (equal). **Oliver P. Love:** Conceptualization (equal); data curation (equal); formal analysis (supporting); funding acquisition (equal); investigation (equal); methodology (equal); project administration (equal); resources (equal); supervision (lead); writing – review and editing (equal). **Kyle H. Elliott:** Conceptualization (equal); data curation (equal); funding acquisition (equal); project administration (equal); resources (equal); writing – review and editing (equal). **H. Grant Gilchrist:** Conceptualization (equal); data curation (equal); funding acquisition (lead); investigation (equal); project administration (equal); resources (equal); writing – review and editing (equal).

## DATA AVAILIBILITY STATEMENT

GPS tracking data will be made available on Movebank, a public repository of tracking data.

## Supporting information


Data S1

Table S1‐S12

Table S13‐S18

Table S19‐S23
