# Supplementary material for: Lower nutritional state and foraging success in an Arctic seabird despite behaviorally flexible responses to environmental change
Source: Ecol Evol. 2023 Apr 20;13(4):e9923. doi: 10.1002/ece3.9923 (PMC10119025; doi:10.1002/ece3.9923)
Supplement: Supplementary file 1 — Data S1 [file ECE3-13-e9923-s003.docx]

**SUPPLEMENTARY FILE**

**Physiology Assays**

***Plasma Triglycerides (TRIG)***

We used a previously validated commercially available assay kit (#TR0100-1KT; Sigma Aldrich, USA; Williams et al. 2007) to measure total and free glycerol from plasma. To measure free glycerol, we ran plasma (8 μL) diluted with ultrapure water (8 μL) in duplicate with control plasma (Sigma-Aldrich, USA), a standard curve of the kit for the glycerol standard (Hennin et al. 2015), and glycerol reagent (240 μL), and measured the solution with a spectrophotometer at 540 nm. To obtain a measurement of total glycerol we added a triglyceride reagent (60 μl) to the solution and measured the solution with a spectrophotometer at 540 nm. We obtained the final TRIG concentration (mmol/L) by taking the difference between free and total glycerol.

***Baseline Corticosterone (bCORT)***

To measure bCORT concentration (ng/mL) we first extracted samples by placing 20 µL of plasma in a tube with 1 mL of distilled water and 5 mL of dichloromethane. We first vortexed the solution, then left the solution for two hours to separate. We transferred the dichloromethane phase into a scintillation vial and placed the vial in a fume hood to evaporate. Following evaporation, we added an assay buffer to rehydrate the samples and we vortexed the samples for 30 seconds. We assayed samples using a commercial enzyme-linked immunoassay kit (EIA; Assay Designs, USA) at a 1:40 dilution in triplicate (Hennin et al. 2015).

***Beta-hydroxybutyrate (B-OH)***

We measured B-OH concentration (mmol/L) in duplicate using a previously-validated kinetic assay (SIGMA, Guglielmo et al. 2002; Lamarre et al. 2017) by adding B-OH dehydrogenase reagent (2 μL) and reagent buffer (2 μL) to plasma (11 μL) or standard (11 μL) then measuring absorbance with a spectrophotometer at 492 nm.

***Non-esterified Fatty Acids (NEFA)***

We used a commercially available assay kit to measure NEFA (NEFA HR2, Wako Diagnostics, USA; Smith et al. 2007; Jeanniard du Dot et al. 2009). We ran all samples in duplicate, adding acyl-CoA synthase, adenosine triphosphate, and CoA to plasma (5 μL) to form acyl-CoA. We obtained NEFA concentrations (mmol/L) by oxidizing samples and measuring the solution with a spectrophotometer at 550 nm.

**References**

Guglielmo, C.G., O’Hara, P.D., and Williams, T.D. 2002. Extrinsic and intrinsic sources of variation in plasma lipid metabolites of free-living Western Sandpipers (Calidris mauri). Auk **119**(2): 437–445.

Hennin, H.L., Legagneux, P., Bêty, J., Williams, T.D., Gilchrist, H.G., Baker, T.M., and Love, O.P. 2015. Pre‑breeding energetic management in a mixed‑strategy breeder. Oecologia **177**: 235–243. doi:10.1007/s00442-014-3145-x.

Jeanniard du Dot, T., Rosen, D.A.S., Richmond, J.P., Kitaysky, A.S., Zinn, S.A., and Trites, A.W. 2009. Changes in glucocorticoids, IGF-I and thyroid hormones as indicators of nutritional stress and subsequent refeeding in Steller sea lions (Eumetopias jubatus). Comp. Biochem. Physiol. Part A **152**(4): 524–534. doi:10.1016/j.cbpa.2008.12.010.

Lamarre, V., Franke, A., Love, O.P., Legagneux, P., and Bêty, J. 2017. Linking pre-laying energy allocation and timing of breeding in a migratory arctic raptor. Oecologia **183**(3): 653–666. doi:10.1007/s00442-016-3797-9.

Smith, S.B., McWilliams, S.R., and Guglielmo, C.G. 2007. Effect of diet composition on plasma metabolite profiles in a migratory songbird. Condor **109**: 48–58.

Williams, T.D., Warnock, N., Takekawa, J.Y., and Bishop, M.A. 2007. Flyway-scale variation in plasma triglyceride levels as an index of refueling rates in spring-migrating Western Sandpipers. Auk **124**(3): 886–897.

**Model Setup**

To assess the impact of sea ice regime on foraging behaviour (fPC1) of murres during the incubation and chick-rearing stages for each colony we fit a model with fPC1 as the response variable and sea ice regime, sex and year (when we had more than two years of data) as the fixed effects. Additionally, for foraging behaviour during the chick-rearing stage for each colony we fit a model with average daily distance as the response variable and sea ice regime, fPC1, and their interaction, along with sex and year as fixed effects. To assess the impact of sea ice regime on average daily energy expenditure for each colony we fit a model with average daily energy expenditure as the response variable with sex, year, sea ice regime, fPC1, and their interaction during the incubation stage and during the chick-rearing stage fixed effects included sex, year, sea ice regime, fPC1, and their interaction, average daily distance and the interaction between sea ice regime and average daily distance.

To assess the impact of sea ice regime on nutritional state and foraging success of murres during the incubation stage for each colony we fit both post-foraging levels and relative change in each of our physiological metrics (mass, bCORT, NEFA, B-OH and TRIG) as response variables with sex, year (when we had more than two years of data), sea ice regime, fPC1 and the interaction between sea ice regime and fPC1 as fixed effects. To assess the impact of sea ice regime on nutritional state and foraging success of murres during the chick-rearing stage at both colonies we fit both post-foraging levels and relative change in each of our physiological metrics (mass, bCORT, NEFA, B-OH and TRIG) as a response variable with sex, year, sea ice regime, fPC1 and the interaction between sea ice regime and fPC1, average daily distance, and the interaction between sea ice regime and average daily distance as fixed effects. For all physiology models, time at the colony before the bird was sampled (TimebfSampling) after returning from a foraging trip was included in the model if it was significant, to account for changes in physiological parameters over time. For all models, GPS deployment duration was included as a fixed effect, if significant, to account for variation in length of GPS deployments.

**Tables**

Table S1. Factor loadings from principal components analysis on foraging metrics (maximum distance, average daily distance, mean trip distance, mean trip duration, and number of trips per day) from GPS deployments on thick-billed murres during the incubation stage at Coats Island, Nunavut and Digges Island, Nunavut. Significant factor loadings are bolded.

Table S2. Factor loadings from principal components analysis on foraging metrics (maximum distance, mean trip distance, mean trip duration, and number of trips per day) from GPS deployments on thick-billed murres during the chick-rearing stage at Coats Island, Nunavut and Digges Island, Nunavut. Significant factor loadings are bolded.

Table S3. Coefficients of intra- and inter- assay variation (%) for plasma triglyceride (TRIG), baseline corticosterone (bCORT), beta-hydroxybutyrate (B-OH), and non-esterified fatty acid (NEFA) assays on plasma samples from 2014, 2015, 2016, 2018, 2019.

Table S4. Summary of inter-colony variation in thick-billed murre GPS deployment duration (Duration; log-scaled), foraging behaviour (fPC1 – maximum distance travelled, average daily distance, mean trip distance, mean trip duration, and number of trips per day), and average daily energy expenditure (DEE; log-scaled) from linear mixed models during the incubation stage at Coats Island and Digges Island, Nunavut.

Table S5. Summary of inter-colony variation in thick-billed murre pre-foraging nutritional state – mass (preMass; g), baseline corticosterone (prebCORT; log-scaled), non-esterified fatty acids (preNEFA; log-scaled), and beta-hydroxybutyrate (preB-OH; log-scaled) from linear mixed models during the incubation stage at Coats Island and Digges Island, Nunavut.

Table S6. Summary of inter-colony variation in thick-billed murre post-foraging nutritional state – mass (postMass; g), beta-hydroxybutyrate (postB-OH; log-scaled), and triglycerides (postTRIG; log-scaled) and foraging success – relative change in beta-hydroxybutyrate (∆B-OH) from linear mixed models during the incubation stage at Coats Island and Digges Island, Nunavut.

Table S7. Summary of inter-colony variation in thick-billed murre pre-foraging nutritional state – triglycerides (preTRIG; log-scaled) and post-foraging nutritional state – baseline corticosterone (postbCORT; log-scaled), and non-esterified fatty acids (postNEFA; log-scaled) from linear models during the incubation stage at Coats Island and Digges Island, Nunavut.

Table S8. Summary of inter-colony variation in thick-billed murre foraging success – relative change in mass (∆Mass), relative change in baseline corticosterone (∆bCORT), relative change in non-esterified fatty acids (∆NEFA), and relative change in triglycerides (∆TRIG) from linear models during the incubation stage at Coats Island and Digges Island, Nunavut.

Table S9. Summary of inter-colony variation in thick-billed murre GPS deployment duration (Duration; log-scaled), foraging behaviour – fPC1 (maximum distance travelled, mean trip distance, mean trip duration, and number of trips per day) and average daily distance (dailyDist), and average daily energy expenditure (DEE) from linear mixed models during the chick-rearing stage at Coats Island and Digges Island, Nunavut.

Table S10. Summary of inter-colony variation in thick-billed murre pre-foraging nutritional state – mass (preMass; g), baseline corticosterone (prebCORT; log-scaled), non-esterified fatty acids (preNEFA; log-scaled), beta-hydroxybutyrate (preB-OH; log-scaled), and triglycerides (preTRIG; log-scaled) from linear mixed models during the chick-rearing stage at Coats Island and Digges Island, Nunavut.

Table S11. Summary of inter-colony variation in thick-billed murre post-foraging nutritional state – mass (postMass; g), baseline corticosterone (postbCORT; log-scaled), non-esterified fatty acids (postNEFA; log-scaled), and foraging success – relative change in mass (∆Mass) and relative change in baseline corticosterone (∆bCORT) from linear mixed models during the chick-rearing stage at Coats Island and Digges Island, Nunavut.

Table S12. Summary of inter-colony variation in thick-billed murre post-foraging nutritional state – beta-hydroxybutyrate (postB-OH; log-scaled) and triglycerides (postTRIG; log-scaled), and foraging success – relative change in non-esterified fatty acids (∆NEFA), relative change in beta-hydroxybutyrate (∆B-OH), and relative change in triglycerides (∆TRIG) from linear models during the chick-rearing stage at Coats Island and Digges Island, Nunavut.

Table S13. Summary of intra-colony variation in thick-billed murre foraging behaviour (fPC1 – maximum distance travelled, average daily distance, mean trip distance, mean trip duration, and number of trips per day), and post-foraging triglyceride levels (log post-TRIG) from linear mixed models during the incubation stage at Digges Island, Nunavut.

Table S14. Summary of intra-colony variation in thick-billed murre average daily energy expenditure (DEE), post-foraging mass (post-Mass), relative change in mass (∆Mass), post-foraging baseline corticosterone levels (log post-bCORT), and relative change in baseline corticosterone (∆bCORT) from linear models during the incubation stage at Digges Island, Nunavut.

Table S15. Summary of intra-colony variation in thick-billed murre relative change in triglycerides (∆TRIG), post-foraging non-esterified fatty acid levels (log post-NEFA), relative change in non-esterified fatty acids (∆NEFA), post-foraging beta-hydroxybutyrate levels (log post-B-OH), and relative change in beta-hydroxybutyrate (∆B-OH) from linear models during the incubation stage at Digges Island, Nunavut.

Table S16. Summary of intra-colony variation in thick-billed murre foraging behaviour (average daily distance – dailyDist and fPC1 – maximum distance travelled, mean trip distance, mean trip duration, and number of trips per day), post-foraging baseline corticosterone levels (log post-bCORT) and post-foraging non-esterified fatty acid levels (log post-NEFA) from linear mixed models during the chick-rearing stage at Digges Island, Nunavut.

Table S17. Summary of intra-colony variation in thick-billed murre average daily energy expenditure (DEE), post-foraging mass (post-Mass), relative change in mass (∆Mass), relative change in baseline corticosterone (∆bCORT), and relative change in non-esterified fatty acids (∆NEFA) from linear models during the chick-rearing stage at Digges Island, Nunavut.

Table S18. Summary of intra-colony variation in thick-billed murre post-foraging triglyceride levels (log post-TRIG), relative change in triglycerides (∆TRIG), post-foraging beta-hydroxybutyrate levels (log post-B-OH), and relative change in beta-hydroxybutyrate (∆B-OH) from linear models during the chick-rearing stage at Digges Island, Nunavut.

Table S19. Summary of intra-colony variation in thick-billed murre foraging behaviour (fPC1 – maximum distance travelled, average daily distance, mean trip distance, mean trip duration, and number of trips per day), average daily energy expenditure (DEE), post-foraging triglyceride levels (log post-TRIG), and relative change in beta-hydroxybutyrate level (∆B-OH) from linear mixed models during the incubation stage at Coats Island, Nunavut.

Table S20. Summary of intra-colony variation in thick-billed murre post-foraging mass (post-Mass), relative change in mass (∆Mass), post-foraging baseline corticosterone levels (log post-bCORT), and relative change in baseline corticosterone (∆bCORT) from linear models during the incubation stage at Coats Island, Nunavut.

Table S21. Summary of intra-colony variation in thick-billed murre relative change in triglycerides (∆TRIG), post-foraging non-esterified fatty acid levels (log post-NEFA), relative change in non-esterified fatty acids (∆NEFA), and post-foraging beta-hydroxybutyrate levels (log post-B-OH) from linear models during the incubation stage at Coats Island, Nunavut.

Table S22. Summary of intra-colony variation in thick-billed murre foraging behaviour (average daily distance – log dailyDist and fPC1 – maximum distance travelled, mean trip distance, mean trip duration, and number of trips per day) and average daily energy expenditure (log DEE), from linear mixed models during the chick-rearing stage at Coats Island, Nunavut.

Table S23. Summary of intra-colony variation in thick-billed murre post-foraging mass (post-Mass) and relative change in mass (∆Mass) from linear models during the chick-rearing stage at Coats Island, Nunavut.

**FIGURES**


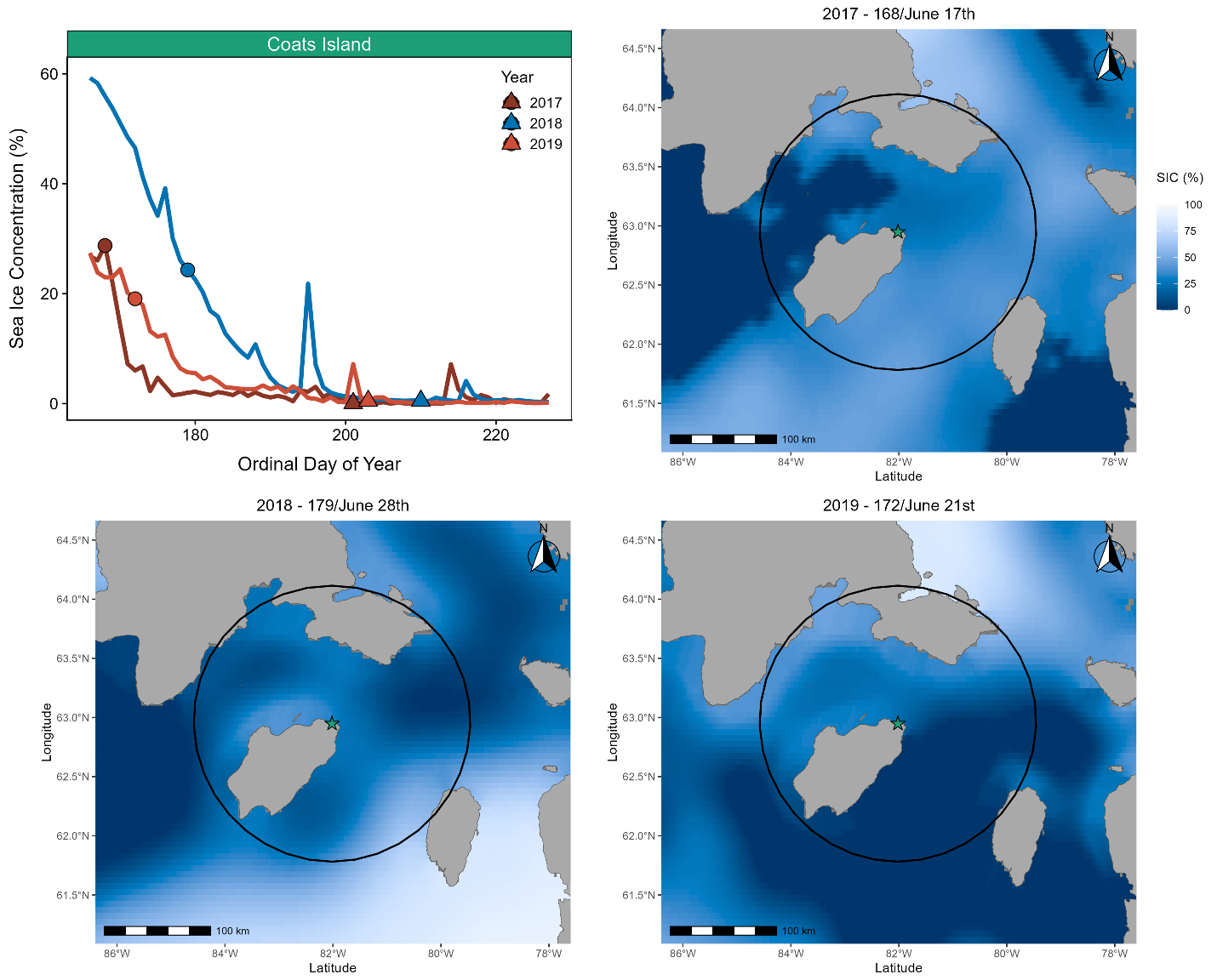


Figure S1. Sea ice concentration (%) throughout the thick-billed murre breeding period (15 June – 15 August) at Coats Island, Nunavut (top left panel), circles depict mean lay dates and triangles depict mean hatch dates respective to study years. Low ice regime years (low sea ice concentration, high sea surface temperarture) are shown in red and light red (2017 and 2019) and high ice regime years (high sea ice concentration, low sea surface temperature) are shown in blue (2018). Maps show sea ice concentration (SIC; %) on median lay dates in 2017 (ordinal day of year 168; June 17th; top right panel), 2018 (ordinal day of year 179; June 28th; bottom left panel), and 2019 (ordinal day of year 172; June 21st; bottom right panel) at Coats Island, Nunavut (turquoise star), black circle indicates the maximum foraging range (130 km) of thick-billed murres at Coats Island.


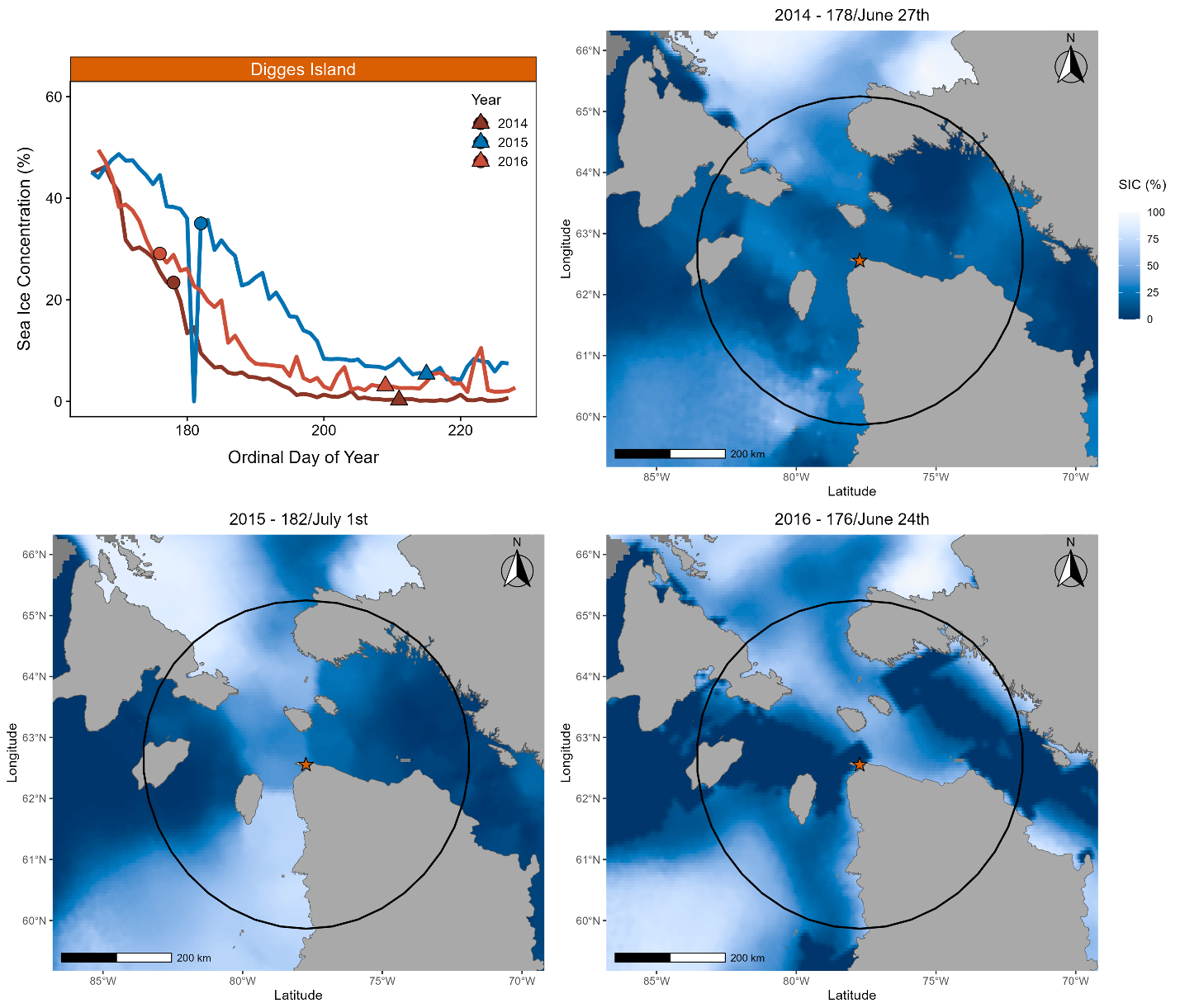


Figure S2. Sea ice concentration (%) throughout the thick-billed murre breeding period (15 June – 15 August) at Digges Island, Nunavut (top left panel), circles depict mean lay dates and triangles depict mean hatch dates respective to study years. Low ice regime years (low sea ice concentration, high sea surface temperarture) are shown in red and light red (2014 and 2016) and high ice regime years (high sea ice concentration, low sea surface temperature) are shown in blue (2015). Maps show sea ice concentration (SIC; %) on median lay dates in 2014 (ordinal day of year 178; June 27^th^; top right panel), 2015 (ordinal day of year 182: July 1st; bottom left panel), and 2016 (ordinal day of year 176; June 24th; bottom right panel) at Digges Island, Nunavut (orange star), black circle indicates the maximum foraging range (300 km) of thick-billed murres at Digges Island.
